# 3-(4-Meth­oxy­benzyl­idene)-1,5-dioxa­spiro­[5.5]undecane-2,4-dione

**DOI:** 10.1107/S1600536810040675

**Published:** 2010-10-20

**Authors:** Wu-Lan Zeng, Jin-Long Suo, Fang-fang Jian

**Affiliations:** a MicroScale Science Institute, Department of Chemistry and Chemical Engineering, Weifang University, Weifang 261061, People’s Republic of China; b MicroScale Science Institute, Weifang University, Weifang 261061, People’s Republic of China

## Abstract

In the title mol­ecule, C_17_H_18_O_5_, which was prepared by the reaction of (*R*)-1,5-dioxaspiro­[5.5]undecane-2,4-dione and 4-meth­oxy­benzaldehyde with ethanol, the 1,3-dioxane ring is in a distorted envelope conformation with the spiro C atom forming the flap. The crystal structure is stabilized by weak inter­molecular C—H⋯O hydrogen bonds.

## Related literature

For background information on spiro-compounds, see: Jiang *et al.* (1998 ▶); Lian *et al.* (2008 ▶); Wei *et al.* (2008 ▶). For a related structure, see: Zeng *et al.* (2009 ▶). For puckering parameters, see: Cremer & Pople (1975 ▶).
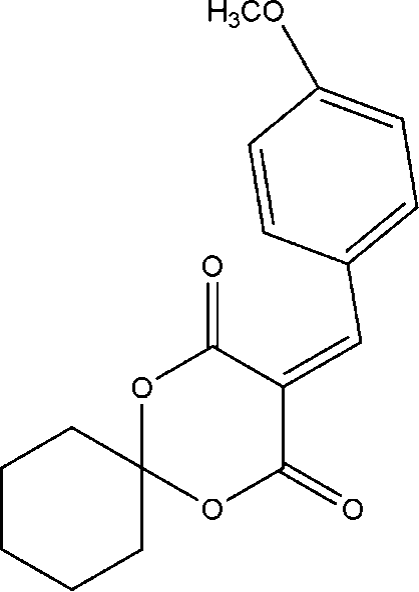


## Experimental

### 

#### Crystal data

C_17_H_18_O_5_
*M* *_r_* = 302.31
Monoclinic, 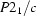

*a* = 15.723 (3) Å
*b* = 10.531 (2) Å
*c* = 9.2029 (18) Å
β = 90.00 (3)°
*V* = 1523.8 (5) Å^3^
*Z* = 4
Mo *K*α radiation
μ = 0.10 mm^−1^
*T* = 293 K
0.25 × 0.16 × 0.10 mm

#### Data collection

Bruker SMART CCD diffractometer
14509 measured reflections
3493 independent reflections
2450 reflections with *I* > 2σ(*I*)
*R*_int_ = 0.057

#### Refinement

*R*[*F* ^2^ > 2σ(*F* ^2^)] = 0.060
*wR*(*F* ^2^) = 0.185
*S* = 1.11
3493 reflections
199 parameters
H-atom parameters constrained
Δρ_max_ = 0.34 e Å^−3^
Δρ_min_ = −0.41 e Å^−3^

### 

Data collection: *SMART* (Bruker, 1997 ▶); cell refinement: *SAINT* (Bruker, 1997 ▶); data reduction: *SAINT*; program(s) used to solve structure: *SHELXS97* (Sheldrick, 2008 ▶); program(s) used to refine structure: *SHELXL97* (Sheldrick, 2008 ▶); molecular graphics: *SHELXTL* (Sheldrick, 2008 ▶); software used to prepare material for publication: *SHELXTL*.

## Supplementary Material

Crystal structure: contains datablocks global, I. DOI: 10.1107/S1600536810040675/hb5678sup1.cif

Structure factors: contains datablocks I. DOI: 10.1107/S1600536810040675/hb5678Isup2.hkl

Additional supplementary materials:  crystallographic information; 3D view; checkCIF report

## Figures and Tables

**Table 1:** Hydrogen-bond geometry (Å, °)

*D*—H⋯*A*	*D*—H	H⋯*A*	*D*⋯*A*	*D*—H⋯*A*
C8—H8*A*⋯O3^i^	0.93	2.58	3.405 (3)	149 (3)

Symmetry code: (i) 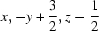.

**Fig. 1.**
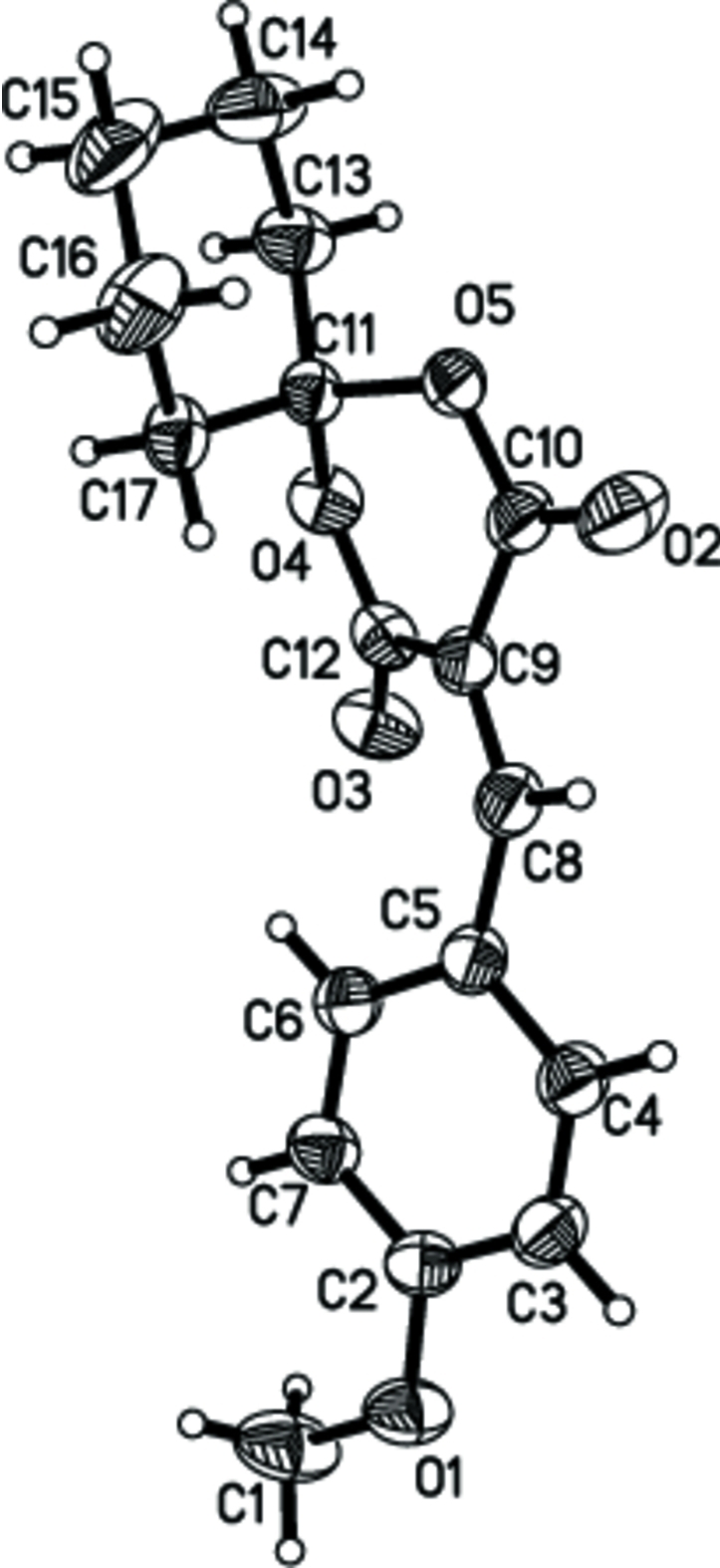
The molecular structure of (I), drawn with 30% probability ellipsoids and spheres of arbritrary size for the H atoms.

## supplementary crystallographic information

### Comment

Spiro compounds are widely used in medicine, catalysis and optical material (Lian *et al.*, 2008; Jiang *et al.*, 1998; Wei *et al.*, 2008) owing to their interesting conformational features. We have recently reported the crystal structure of (Z)-3-(3-phenylallylidene)-1,5-dioxaspiro[5.5]undecane-2,4-dione (Zeng *et al.* 2009). As part of our ongoing studies on new spiro compounds with potentially higher bioactivity, the title compound, (I) (Fig. 1), has been synthesized and its structure is reported here.

The 1,3-dioxane ring is in a distored envelope conformation with atom C11 atom common to the cyclohexane forming the flap. The cyclohexane exists in a distorted chair comformation, with the puckering parameters Q=0.552Å, theta=175.1°, Phi=39.2°; The crystal structure is stabilized by weak intra and intermolecular C—H···O hydrogen bonds (Table 1).

### Experimental

A mixture of malonic acid (6.24 g, 0.06 mol) and acetic anhydride(9 ml) in strong sulfuric acid (0.25 ml) was stirred with water at 303 K. After dissolving, cyclohexanone (5.88 g, 0.06 mol) was added dropwise into solution for 1 h. The reaction was allowed to proceed for 4 h. The mixture was cooled and filtered, and then an ethanol solution of 4-methoxybenzaldehyde (8.16 g, 0.06 mol) was added. The solution was then filtered and concentrated.  Colourless blocks of (I) were obtained by evaporation of an petroleum ether-ethylacetate (3:1 *v*/*v*) solution at room temperature over a period of one week.

### Refinement

The H atoms were placed in calculated positions (C—H = 0.93–0.97 Å), and refined as riding with *U*_iso_(H) = 1.2*U*_eq_(C) or 1.5U_eq_(methyl C).

### Crystal data

**Table:**

C_17_H_18_O_5_	*F*(000) = 640
*M**_r_* = 302.31	*D*_x_ = 1.318 Mg m^−^^3^
Monoclinic, *P*2_1_/*c*	Mo *K*α radiation, λ = 0.71073 Å
Hall symbol: -P 2ybc	Cell parameters from 2450 reflections
*a* = 15.723 (3) Å	θ = 3.2–27.5°
*b* = 10.531 (2) Å	µ = 0.10 mm^−^^1^
*c* = 9.2029 (18) Å	*T* = 293 K
β = 90.00 (3)°	Block, colorless
*V* = 1523.8 (5) Å^3^	0.25 × 0.16 × 0.10 mm
*Z* = 4	

### Data collection

**Table:**

Bruker SMART CCD diffractometer	2450 reflections with *I* > 2σ(*I*)
Radiation source: fine-focus sealed tube	*R*_int_ = 0.057
graphite	θ_max_ = 27.5°, θ_min_ = 3.2°
phi and ω scans	*h* = −20→20
14509 measured reflections	*k* = −11→13
3493 independent reflections	*l* = −11→11

### Refinement

**Table:**

Refinement on *F*^2^	Primary atom site location: structure-invariant direct methods
Least-squares matrix: full	Secondary atom site location: difference Fourier map
*R*[*F*^2^ > 2σ(*F*^2^)] = 0.060	Hydrogen site location: inferred from neighbouring sites
*wR*(*F*^2^) = 0.185	H-atom parameters constrained
*S* = 1.11	*w* = 1/[σ^2^(*F*_o_^2^) + (0.0988*P*)^2^ + 0.1666*P*] where *P* = (*F*_o_^2^ + 2*F*_c_^2^)/3
3493 reflections	(Δ/σ)_max_ < 0.001
199 parameters	Δρ_max_ = 0.34 e Å^−^^3^
0 restraints	Δρ_min_ = −0.41 e Å^−^^3^

### Special details

**Table:**

Geometry. All e.s.d.'s (except the e.s.d. in the dihedral angle between two l.s. planes) are estimated using the full covariance matrix. The cell e.s.d.'s are taken into account individually in the estimation of e.s.d.'s in distances, angles and torsion angles; correlations between e.s.d.'s in cell parameters are only used when they are defined by crystal symmetry. An approximate (isotropic) treatment of cell e.s.d.'s is used for estimating e.s.d.'s involving l.s. planes.
Refinement. Refinement of *F*^2^ against ALL reflections. The weighted *R*-factor *wR* and goodness of fit *S* are based on *F*^2^, conventional *R*-factors *R* are based on *F*, with *F* set to zero for negative *F*^2^. The threshold expression of *F*^2^ > σ(*F*^2^) is used only for calculating *R*-factors(gt) *etc*. and is not relevant to the choice of reflections for refinement. *R*-factors based on *F*^2^ are statistically about twice as large as those based on *F*, and *R*- factors based on ALL data will be even larger.

### Fractional atomic coordinates and isotropic or equivalent isotropic displacement parameters (Å^2^)

**Table:**

	*x*	*y*	*z*	*U*_iso_*/*U*_eq_	
O5	0.35323 (7)	0.60512 (11)	0.18736 (14)	0.0621 (4)	
O4	0.27576 (8)	0.50924 (12)	0.37464 (12)	0.0613 (4)	
O3	0.14427 (8)	0.56348 (16)	0.42086 (13)	0.0767 (4)	
C12	0.20204 (11)	0.56349 (17)	0.33460 (18)	0.0573 (4)	
O1	−0.22145 (8)	0.60711 (16)	0.16227 (16)	0.0799 (5)	
C9	0.20107 (11)	0.62557 (17)	0.1912 (2)	0.0591 (4)	
C11	0.34102 (10)	0.49138 (15)	0.26925 (17)	0.0520 (4)	
C5	0.04075 (11)	0.64170 (17)	0.13577 (19)	0.0596 (4)	
O2	0.29512 (10)	0.73591 (18)	0.0312 (2)	0.1155 (7)	
C2	−0.13524 (11)	0.61345 (19)	0.15722 (19)	0.0612 (4)	
C17	0.31883 (13)	0.38211 (18)	0.1710 (2)	0.0663 (5)	
H17A	0.2690	0.4036	0.1141	0.080*	
H17B	0.3054	0.3080	0.2293	0.080*	
C4	−0.01498 (12)	0.72360 (19)	0.0631 (2)	0.0696 (5)	
H4A	0.0072	0.7881	0.0054	0.084*	
C6	0.00472 (12)	0.54299 (19)	0.2157 (2)	0.0661 (5)	
H6A	0.0402	0.4856	0.2628	0.079*	
C13	0.42224 (12)	0.4727 (2)	0.3519 (2)	0.0744 (6)	
H13A	0.4141	0.4065	0.4239	0.089*	
H13B	0.4364	0.5505	0.4027	0.089*	
C8	0.13114 (12)	0.66335 (18)	0.1175 (2)	0.0669 (5)	
H8A	0.1438	0.7143	0.0378	0.080*	
C7	−0.08152 (12)	0.5284 (2)	0.2265 (2)	0.0668 (5)	
H7A	−0.1040	0.4617	0.2803	0.080*	
C10	0.28435 (12)	0.66098 (19)	0.1279 (2)	0.0707 (5)	
C3	−0.10110 (13)	0.7112 (2)	0.0747 (2)	0.0725 (5)	
H3A	−0.1368	0.7681	0.0274	0.087*	
C15	0.4723 (2)	0.3234 (3)	0.1548 (4)	0.1182 (11)	
H15A	0.5187	0.3067	0.0880	0.142*	
H15B	0.4637	0.2483	0.2139	0.142*	
C14	0.49532 (14)	0.4363 (3)	0.2527 (3)	0.1001 (9)	
H14A	0.5445	0.4144	0.3112	0.120*	
H14B	0.5104	0.5085	0.1926	0.120*	
C16	0.39229 (19)	0.3516 (3)	0.0700 (3)	0.1034 (9)	
H16A	0.3777	0.2787	0.0107	0.124*	
H16B	0.4023	0.4231	0.0058	0.124*	
C1	−0.25940 (14)	0.5094 (3)	0.2471 (3)	0.1045 (9)	
H1A	−0.3202	0.5159	0.2409	0.157*	
H1B	−0.2420	0.5182	0.3466	0.157*	
H1C	−0.2416	0.4280	0.2110	0.157*	

### Atomic displacement parameters (Å^2^)

**Table:**

	*U*^11^	*U*^22^	*U*^33^	*U*^12^	*U*^13^	*U*^23^
O5	0.0527 (7)	0.0513 (7)	0.0822 (8)	−0.0017 (5)	0.0135 (6)	0.0140 (5)
O4	0.0618 (7)	0.0751 (8)	0.0470 (6)	−0.0023 (6)	0.0115 (5)	−0.0021 (5)
O3	0.0619 (8)	0.1095 (12)	0.0587 (7)	−0.0099 (7)	0.0195 (6)	−0.0150 (7)
C12	0.0573 (9)	0.0596 (10)	0.0549 (9)	−0.0089 (8)	0.0141 (7)	−0.0117 (7)
O1	0.0530 (8)	0.1061 (12)	0.0808 (9)	−0.0026 (7)	0.0057 (6)	0.0112 (8)
C9	0.0532 (9)	0.0508 (9)	0.0732 (11)	0.0017 (7)	0.0182 (8)	0.0042 (7)
C11	0.0554 (9)	0.0501 (9)	0.0506 (8)	−0.0030 (7)	0.0110 (7)	0.0037 (6)
C5	0.0575 (10)	0.0563 (10)	0.0650 (10)	0.0027 (8)	0.0121 (7)	0.0050 (7)
O2	0.0713 (10)	0.1084 (13)	0.1668 (16)	0.0208 (9)	0.0394 (10)	0.0879 (12)
C2	0.0524 (9)	0.0747 (12)	0.0563 (9)	0.0010 (8)	0.0056 (7)	−0.0017 (8)
C17	0.0790 (13)	0.0524 (10)	0.0675 (11)	0.0010 (8)	0.0074 (9)	−0.0063 (8)
C4	0.0639 (11)	0.0664 (12)	0.0787 (12)	0.0033 (9)	0.0108 (9)	0.0185 (9)
C6	0.0606 (11)	0.0611 (11)	0.0765 (12)	−0.0023 (8)	−0.0002 (8)	0.0152 (9)
C13	0.0626 (11)	0.0894 (15)	0.0710 (11)	−0.0060 (10)	−0.0017 (9)	0.0195 (10)
C8	0.0621 (11)	0.0569 (10)	0.0816 (12)	0.0061 (8)	0.0200 (9)	0.0130 (8)
C7	0.0622 (11)	0.0716 (12)	0.0666 (10)	−0.0104 (9)	0.0009 (8)	0.0157 (9)
C10	0.0582 (10)	0.0566 (11)	0.0972 (14)	0.0080 (8)	0.0229 (9)	0.0234 (9)
C3	0.0640 (12)	0.0718 (12)	0.0816 (12)	0.0103 (9)	0.0032 (9)	0.0159 (10)
C15	0.110 (2)	0.105 (2)	0.140 (2)	0.0499 (18)	0.0472 (19)	0.0149 (19)
C14	0.0599 (12)	0.129 (2)	0.1108 (18)	0.0150 (13)	0.0135 (12)	0.0447 (18)
C16	0.115 (2)	0.0978 (19)	0.0974 (16)	0.0332 (16)	0.0266 (15)	−0.0256 (14)
C1	0.0627 (13)	0.159 (3)	0.0916 (16)	−0.0262 (14)	0.0035 (11)	0.0349 (16)

### Geometric parameters (Å, °)

**Table:**

O5—C10	1.348 (2)	C4—C3	1.365 (3)
O5—C11	1.4281 (19)	C4—H4A	0.9300
O4—C12	1.344 (2)	C6—C7	1.368 (3)
O4—C11	1.4245 (19)	C6—H6A	0.9300
O3—C12	1.2064 (19)	C13—C14	1.516 (3)
C12—C9	1.473 (3)	C13—H13A	0.9700
O1—C2	1.358 (2)	C13—H13B	0.9700
O1—C1	1.423 (3)	C8—H8A	0.9300
C9—C8	1.352 (3)	C7—H7A	0.9300
C9—C10	1.481 (2)	C3—H3A	0.9300
C11—C13	1.499 (3)	C15—C16	1.509 (4)
C11—C17	1.504 (2)	C15—C14	1.535 (5)
C5—C6	1.394 (3)	C15—H15A	0.9700
C5—C4	1.400 (3)	C15—H15B	0.9700
C5—C8	1.449 (3)	C14—H14A	0.9700
O2—C10	1.201 (2)	C14—H14B	0.9700
C2—C7	1.386 (3)	C16—H16A	0.9700
C2—C3	1.387 (3)	C16—H16B	0.9700
C17—C16	1.517 (3)	C1—H1A	0.9600
C17—H17A	0.9700	C1—H1B	0.9600
C17—H17B	0.9700	C1—H1C	0.9600
			
C10—O5—C11	118.18 (13)	H13A—C13—H13B	107.9
C12—O4—C11	119.39 (13)	C9—C8—C5	133.89 (17)
O3—C12—O4	117.97 (17)	C9—C8—H8A	113.1
O3—C12—C9	125.58 (18)	C5—C8—H8A	113.1
O4—C12—C9	116.32 (14)	C6—C7—C2	119.86 (18)
C2—O1—C1	118.23 (18)	C6—C7—H7A	120.1
C8—C9—C12	126.06 (16)	C2—C7—H7A	120.1
C8—C9—C10	116.61 (16)	O2—C10—O5	118.29 (17)
C12—C9—C10	117.06 (16)	O2—C10—C9	125.55 (18)
O4—C11—O5	110.20 (13)	O5—C10—C9	116.15 (15)
O4—C11—C13	106.60 (14)	C4—C3—C2	119.86 (18)
O5—C11—C13	105.28 (14)	C4—C3—H3A	120.1
O4—C11—C17	110.06 (14)	C2—C3—H3A	120.1
O5—C11—C17	110.83 (14)	C16—C15—C14	110.4 (2)
C13—C11—C17	113.69 (16)	C16—C15—H15A	109.6
C6—C5—C4	117.22 (17)	C14—C15—H15A	109.6
C6—C5—C8	125.26 (17)	C16—C15—H15B	109.6
C4—C5—C8	117.48 (16)	C14—C15—H15B	109.6
O1—C2—C7	124.10 (17)	H15A—C15—H15B	108.1
O1—C2—C3	116.19 (17)	C13—C14—C15	111.7 (2)
C7—C2—C3	119.70 (17)	C13—C14—H14A	109.3
C11—C17—C16	110.71 (19)	C15—C14—H14A	109.3
C11—C17—H17A	109.5	C13—C14—H14B	109.3
C16—C17—H17A	109.5	C15—C14—H14B	109.3
C11—C17—H17B	109.5	H14A—C14—H14B	107.9
C16—C17—H17B	109.5	C15—C16—C17	111.1 (2)
H17A—C17—H17B	108.1	C15—C16—H16A	109.4
C3—C4—C5	121.64 (18)	C17—C16—H16A	109.4
C3—C4—H4A	119.2	C15—C16—H16B	109.4
C5—C4—H4A	119.2	C17—C16—H16B	109.4
C7—C6—C5	121.66 (18)	H16A—C16—H16B	108.0
C7—C6—H6A	119.2	O1—C1—H1A	109.5
C5—C6—H6A	119.2	O1—C1—H1B	109.5
C11—C13—C14	111.92 (18)	H1A—C1—H1B	109.5
C11—C13—H13A	109.2	O1—C1—H1C	109.5
C14—C13—H13A	109.2	H1A—C1—H1C	109.5
C11—C13—H13B	109.2	H1B—C1—H1C	109.5
C14—C13—H13B	109.2		

### Hydrogen-bond geometry (Å, °)

**Table:**

*D*—H···*A*	*D*—H	H···*A*	*D*···*A*	*D*—H···*A*
C8—H8A···O3^i^	0.93	2.58	3.405 (3)	149 (3)

Symmetry codes: (i) *x*, −*y*+3/2, *z*−1/2.
